# Structure prediction for the helical skeletons detected from the low resolution protein density map

**DOI:** 10.1186/1471-2105-11-S1-S44

**Published:** 2010-01-18

**Authors:** Kamal Al Nasr, Weitao Sun, Jing He

**Affiliations:** 1 Department of Computer Science, Old Dominion University, Norfolk, VA 23529, USA; 2 Zhou Pei-Yuan Center for Applied Mathematics, Tsinghua University, Beijing, 100084, PR China; 3 Department of Computer Science, New Mexico State University, Las Cruces, NM, 88003, USA

## Abstract

**Background:**

The current advances in electron cryo-microscopy technique have made it possible to obtain protein density maps at about 6-10 Å resolution. Although it is hard to derive the protein chain directly from such a low resolution map, the location of the secondary structures such as helices and strands can be computationally detected. It has been demonstrated that such low-resolution map can be used during the protein structure prediction process to enhance the structure prediction.

**Results:**

We have developed an approach to predict the 3-dimensional structure for the helical skeletons that can be detected from the low resolution protein density map. This approach does not require the construction of the entire chain and distinguishes the structures based on the conformation of the helices. A test with 35 low resolution density maps shows that the highest ranked structure with the correct topology can be found within the top 1% of the list ranked by the effective energy formed by the helices.

**Conclusion:**

The results in this paper suggest that it is possible to eliminate the great majority of the bad conformations of the helices even without the construction of the entire chain of the protein. For many proteins, the effective contact energy formed by the secondary structures alone can distinguish a small set of likely structures from the pool.

## Background

X-ray crystallography is a well known biophysical technique to determine the tertiary structure of proteins. Given a protein crystal of good quality, this technique can often generate the electron density map to higher than 4 Å resolution from the X-ray diffraction data. The backbone of the protein can often be derived from such density maps using crystallography software [1]. However, if the electron density map has low resolution, such as 6-10 Å, the typical software can not derive the backbone of the protein since the characteristics of amino acids are not well resolved at this resolution. Low resolution protein density map are more and more abundant as the electron cryomicroscopy technique advances [2-6]. This technique does not require growing protein crystals which is often a limiting factor for structure determination using X-ray crystallography technique [2].

**Figure 1:**
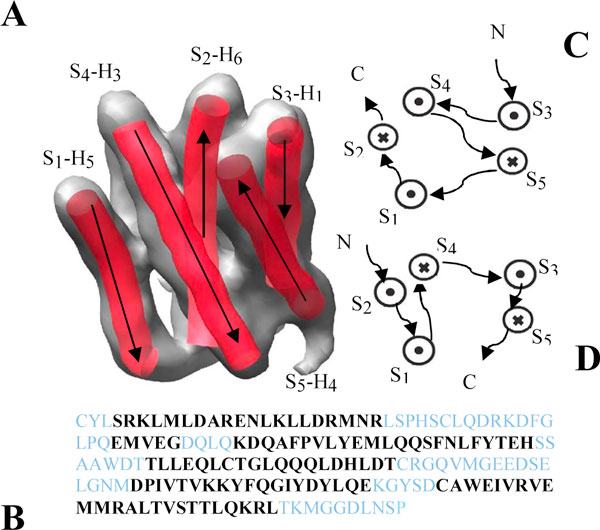
**Helical skeletons and topologies**. (A) The density map (grey) was simulated to 10 Å resolution using protein 1B5L from the Protein Data Bank (PDB). The helical skeletons (red S_1 _to S_5_) were detected using Helix Tracer [8]. (B) The helix segments are highlighted (black) on the proteins sequence. Two alternative topologies are shown as diagrams in (C) correct and (D) wrong topology, in which the N to C direction for the loop (arrow) and for the skeleton (cross and dot) is labelled. The true assignment is labelled on the skeleton with H_1 _being the first helix segment on the protein sequence.

At low resolution, the location and orientation of the secondary structures such as helices and β-sheets can be computationally identified [7-10]. It is also possible to derive the β-strands computationally [11]. Since the loop densities are not well resolved, the connection of the adjacent secondary structure elements is often not available. Figure 1 shows an example of a density map and the computationally detected helical skeletons using Helix Tracer [8]. In this case, Helix Tracer was able to detect five skeletons that represent the electron density of five helices. The shortest helix which has five amino acids was not detectable by Helix Tracer. Each skeleton can be represented by the coordinates of the central axis of the helix. However, it is not known which segment of the protein sequence corresponds to which skeleton. The problem studied in this paper is how to predict the structure for the helical skeletons. Once the structure of the skeletons are predicted, loops can be added using our previous method [12][13] or other existing loop closure methods [14-20].

Given a protein sequence, the location of secondary structures on the sequence can be roughly predicted using the existing secondary structure prediction (SSP) methods. Such methods can generally predict the secondary structures to about 70-80% accuracy [21-23]. It is possible to derive the native topology for the skeletons by mapping the sequence segments obtained from SSP to the skeletons detected from the density map [24-26]. Secondary structure topology in this paper refers to the order and the direction of the secondary structures such as helices and strands with respect to the protein sequence. For a protein with *N *helices and *M *strands, there are (*N*!*2*^*N*^)(*M*!*2*^*M*^) different topologies if there are *N *helical skeletons and *M *strand skeletons. This is because there are *N*! different orders for assigning *N *helices and 2 directions to assign each helix. When the number of skeletons is not the same as the number of the sequence segments, the number of topologies is  where *K *is the number of helical skeletons assuming *K *≤ *N*, which is often true when only the reliably detected skeletons are considered for mapping. This paper only explores the structure prediction problem for the helical skeletons. We have not extended the work to the skeletons of β-strands.

It has been an active research area to use a combination of structure prediction and the protein density map to derive the tertiary structure for the proteins. One approach can be considered as "sequence initiated". It uses the existing comparative modeling [27,28] or ab initio structure prediction methods [29,30] to generate the initial possible conformations of the protein and use the density map to enhance the evaluation of the conformations. Another approach can be considered as "combined density and sequence initiated". It builds the initial conformations using both the density and sequence information. This approach has suggested that the native topology of the secondary structures can be predicted near the top of the list [24-26].

Our previous work has shown that if the Cα atoms of the secondary structures are known, the native secondary structure topology can be ranked near top of the list even without modeling the loops [25,26]. In this paper, we started with the protein density map instead of the assumption of the locations for the Cα atoms. We present a method that predicts the tertiary structure for the helical skeletons without building the entire chain of the protein. Our test using 35 proteins shows that a near native structure is ranked near the top of the list for the helical skeletons in the density map.

Since our method predicts the structure for helices without building the entire chain, we explored the perspective of applying it to the structure prediction in large proteins in this paper. Although comparative modeling method can be used to predict the structure of the large proteins, it requires the template structures that share certain level of similarity to the target structure [31,32]. Instead of constructing the entire chain which is almost impossible for a large protein, we will show the preliminary results of a novel approach to predict the structure of multiple local regions where characteristic helical skeletons are located.

## Results and discussion

**Figure 2:**
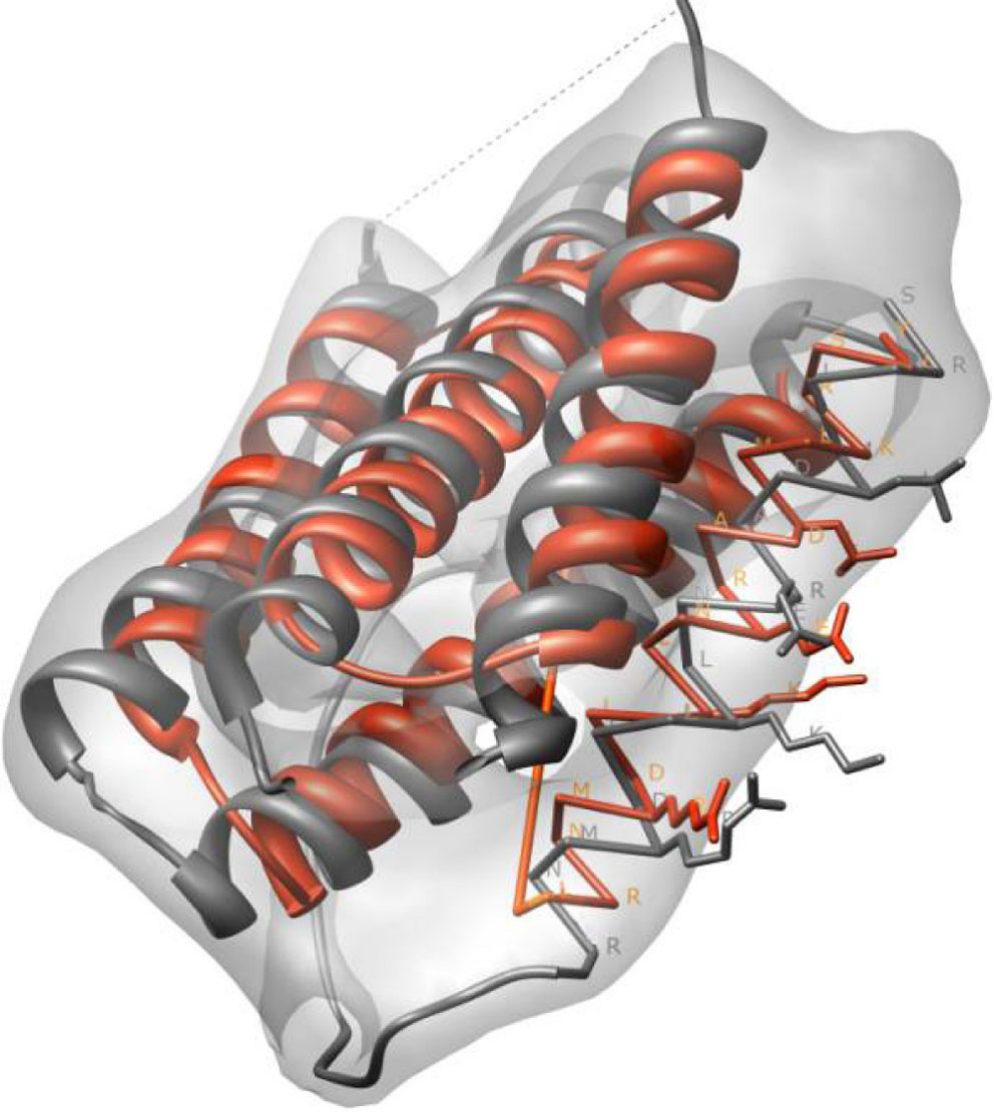
**The highest ranked structure with the correct topology for **1B5L** (PDB ID)**. The native structure (grey ribbon) and the predicted structure (red ribbon) were superimposed on the protein density map. In the predicted structure, the connection between the two helices is simply drawn as a straight line that is smoothed by the ribbon representation. The amino acid labels and the side chains are shown for one of the five helices. The dotted line (grey) represents the missing loop in the native structure.

Given the protein density map at 6-10 Å resolution and its primary structure, our method generates a list of possible 3-dimentional structures for the helices of the protein. Figure 2 shows an example of the predicted structure for the helix skeletons detected from the 10 Å resolution protein density map. In this case, Helix Tracer detected five of the six helices in this protein (1B5L, the 34^th ^protein in Table 1). In theory, there are totally  = 23040 different topologies, with each one representing a specific order and direction of the skeletons [25,26]. After distance and length screening there were 438 valid topologies (Table 1, row 34). For each valid topology, 500 structures were generated using simulated annealing to sample the freedom from (*S*_1_, θ_1_), (*S*_2_, θ_2_), ..., (*S*_5_, θ_5_), (details in Methods section). The translation along the helix axis was set to zero for simplicity. The predicted structures were sorted by the effective contact energy formed by the helices [25]. The highest ranked structure with the correct topology (red in Figure 2) is the 759^th ^out of 219000 structures (Table 1, row 34). It has a backbone Root-Mean-Square-Deviation (RMSD) of 5.44 Å from the native protein. The RMSD was calculated for the helix portion of the chain that was constructed by our program. Note that our method predicts the helix portion of the chain without building the loops; the predicted structure does not have the information about the loops. The two adjacent helices were simply connected with a straight line between the last C atom of the first helix and the first N atom of the next helix (Figure 2). The amino acid names were shown for one of the five helices (Figure 2). For viewing clarity, certain constructed side chains were shown for that helix. It can be seen that the sequence segment, the direction of the assignment are correct for this helix when the predicted helix is compared to its native peer. We noticed that the perfect helix model has introduced error in the predicted structure, since helices are often not perfectly straight and contain slightly different dihedral angles (data not shown).

**Table 1:** The test of the structure prediction for the helical skeletons

No	ID	#AA^a^	#hlces^b^	#sticks^c^	#Possible Topologies^d^	#Valid Topologies^e^	#Generated structures^f^	Rank^g^	RMSD^h^	Prct^i^
1	1DP3	55	3	3	48	6	3000	10	4.78	0.33%

2	1A2T	149	3	3	48	32	16000	15	3.72	0.09%

3	1AIL	73	3	3	48	16	8000	3	3.96	0.04%

4	1BUU	168	3	3	48	16	8000	27	4.67	0.34%

5	1lRE	81	3	3	48	18	9000	10	4.55	0.11%

6	1BR0	120	3	3	48	4	2000	4	11.17	0.20%

7	1B67	68	3	3	48	14	7000	17	4.03	0.24%

8	1AYI	87	4	3	192	48	24000	93	3.8	0.39%

9	1BEA	127	4	3	192	160	80000	572	4.75	0.72%

10	1GXG	85	4	3	192	40	20000	12	2.8	0.06%

11	1NO1	126	4	3	192	120	60000	42	4.24	0.07%

12	2EZH	75	4	3	192	104	52000	47	4.2	0.09%

13	1A3C	181	4	3	192	48	24000	110	3.57	0.46%

14	1A32	88	4	3	192	40	20000	9	3.69	0.05%

15	1PZ4	116	4	3	192	72	36000	147	7.3	0.41%

16	1LIH	164	4	3	192	124	62000	114	5.48	0.18%

17	1DXS	80	5	3	480	450	225000	30	3.51	0.01%

18	1SU0	159	5	3	480	96	48000	60	6.69	0.13%

19	1BO9	73	6	3	960	438	219000	35	3.43	0.02%

20	1JW2	72	4	4	384	66	33000	31	4.26	0.09%

21	1I2T	61	4	4	384	64	32000	21	5.58	0.07%

22	1CCD	77	4	4	384	20	10000	3	4.75	0.03%

23	2PSR	100	5	4	1920	468	234000	339	4.75	0.14%

24	1A7D	118	6	4	5760	139	69500	144	5.33	0.21%

25	2LIS	136	6	4	5760	144	72000	8	4.84	0.01%

26	1ALU	186	6	4	5760	419	209500	288	7.22	0.14%

27	1HXI	121	6	4	5760	400	200000	17	3.78	0.01%

28	1JMW	146	6	4	5760	304	152000	129	5.25	0.08%

29	1AA2	108	7	4	13440	768	384000	3599	4.15	0.94%

30	1BVC	153	8	4	26880	1215	607500	8	5.59	0.00%

31	1BZ4	144	5	5	3840	16	8000	31	4.91	0.39%

32	1AEP	161	5	5	3840	157	78500	204	5.3	0.26%

33	1DUS	194	6	5	23040	3840	1920000	2179	4.44	0.11%

34	1B5L	172	6	5	23040	438	219000	759	5.44	0.35%

35	1FLP	142	7	6	322560	7734	3867000	4707	4.65	0.12%

a: the number of amino acids in the protein

b: the number of helices in the protein

c: the number of skeletons detected by Helix Tracer

d: the number of all possible topologies

e: the number of valid topologies after applying distance and length screening

f: the number of structures generated for all valid topologies

g: the highest rank of the structure that has the correct topology

h: the Root Mean Square Deviation (RMSD) of Cá atoms of the structure that has the highest rank with the correct topology

i: the percentage of the highest rank among all generated structures

To test the performance of our method, we generated thirty-five density maps at 10 Å resolution [33] using the native structures from the PDB. The proteins were randomly selected among the proteins that have three to seven helices (Table 1 column 4). The total number of possible topologies  is shown in the 6^th ^column. It appears that the distance and the length screening are generally effective to reduce the number of topologies (column 6 and 7). However, this reduction is protein dependent. For some proteins, it only reduces less than 10% of the topologies (1DXS, row 17), and for other proteins, it reduces more than 80% (1JW2, 20^th ^row). This is expected since the distance screening can only reduce the topologies in which the loops appear to be short in amino acid sequence but long in the density map and not the other way around. The structures were ranked by the contact energy formed by the constructed helices and not including the loops. The highest rank of the structure that has the correct topology is listed in column 9 (Table 1). Our previous study has shown that if the backbone coordinates are fixed, the correct topology can generally be located at the top 25% of the list that is ranked by the effective contact energy [25]. In this study we relaxed the requirement of fixing the backbone coordinates and built the possible backbones from the central helical axis. This involves the sampling of the rotation and translation freedom about the helix axis. Our simulated annealing test in this paper suggests that a near-native helical structure can be found within the top 1% of the structures generated (column 11 Table 1).

**Figure 3:**
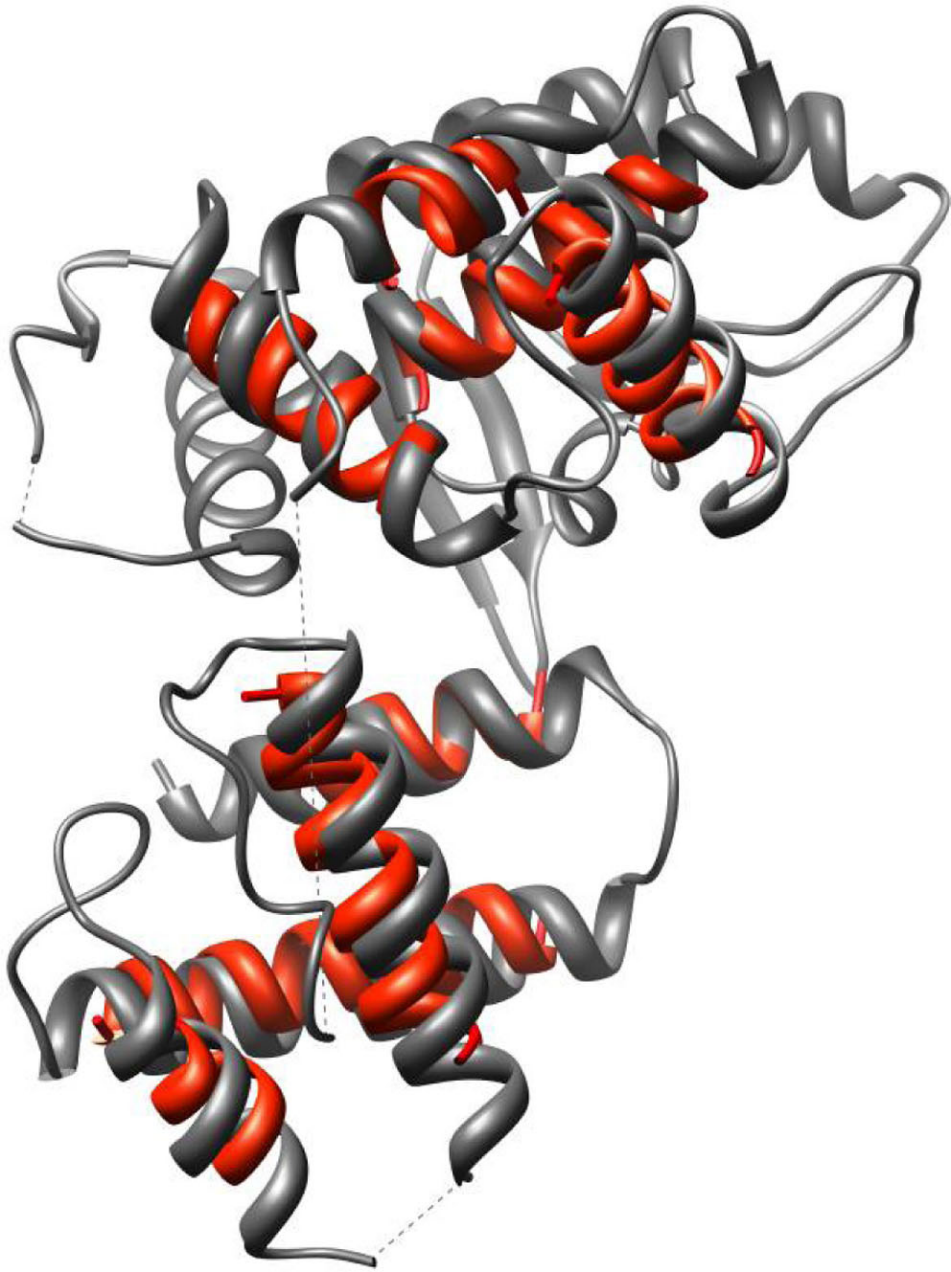
**The predicted structure for eight of the fourteen helices in two regions**. The native structure (PDB ID: 1A0P in grey) and the highest ranked structure with the correct topology (red).

Since our method predicts the structure for the helical skeletons without building the entire chain, we explored the possibility of applying it to large proteins at multiple local regions. We performed a test on two proteins that have 290 and 322 amino acids respectively (Table 2). For each protein, we generated their density map at 10 Å resolution and used the Helix Tracer to detect the skeletons. We selected two local regions with closely associated skeletons and wanted to see how well our program can predict a near native structure for the local regions without building the entire chain of the protein. Each local region consists of four helical skeletons. The structures constructed for each local region were ranked by their effective contact energy. The highest ranked structure that has the correct topology is at the 10448^th ^of the 6973800 pool of structures generated for the first local region (1A0P_G1, the 2^nd ^row of Table 2). The structure for region G1 has a backbone RMSD of 3.96 Å when it is compared with its native peer (Figure 3 and Table 2). It is ranked at the top 0.15% in the pool of structures for this region. The two local regions we selected have no common skeletons, although they may have in principle. We simply combined the ranked list of structures for the first local region (G1) with that for the second local region (G2). Since each list is developed independently from the other, the conflicting assignments need to be eliminated when the two lists are combined. A conflicting assignment is such that the same segment of the sequence is assigned to both a skeleton in the first region and a skeleton in the second region. After this screening, the highest ranked structure with the correct topology (red ribbon in Figure 3) for eight skeletons was ranked the 3741775^th ^of a pool of 5.9E+13 structures, about the top 0% of the list.

**Table 2:** Structure prediction of the local regions in two large proteins

ID	#AA^a^	#hlces^b^	#sticks^c^	#Possible Topologies^d^	#Valid Topologies^e^	#Generated structures^f^	Rank^g^	RMSD^h^	Prct^i^
1A0P_G1		14	4	384384	69738	6973800	10448	3.96	0.15%

1A0P_G2		14	4	384384	84733	8473300	14673	4.11	0.17%

1A0P	290	14	8			5.9E+13	3741775		0%

1WQ3_G1		20	4	1860480	184255	18425500	40485	6.47	0.22%

1WQ3_G2		20	4	1860480	280708	28070800	18412	4.65	0.07%

1WQ3	322	20	8			5.17E+14	32104299		0%

a: the number of amino acids in the protein

b: the number of helices in the protein

c: the number of skeletons used for structure prediction in the region

d: the number of all possible topologies in the region

e: the number of valid topologies after applying distance and length screening

f: the number of structures generated in the region or the total number of the structures evaluated for two regions

g: the highest rank of the structure with the correct topology among the generated structures

h: the RMSD of the highest ranked structure with the correct topology among the generated structures

i: the percentage of the rank for the structure with the highest rank and the correct topology

Our exploratory test about the local regions of large proteins used minimum rules to eliminate the impossible structures. We expect that more geometrical rules can be used to enhance the ranking of the near-native structure. This paper suggests that a near-native structure for the helical skeletons can be found near the top of the list ranked by the effective contact energy. In order to generate a few most likely structures, additional evaluation is needed involving statistical analysis of the likely structures, refinement of the structures and using additional information from the density map.

## Conclusion

Our previous work has shown that if the Cα atoms of the helices are fixed, the correct topology can be ranked within the top 25% of the list ranked by the effective energy that is formed by the helices [25,26]. This approach does not involve the construction of the loops, yet is still able to distinguish most of the bad structures. In this paper, we have relaxed the assumption of the fixed locations for Cα atoms. We have developed a method to construct the backbone and the side chains using the central helical axis detected from the low resolution density map. We used a combination of approaches in this paper to work with the even larger solution space. Such approaches include the newly developed parallel simulated annealing process, the distance and length screening and the incorporation of more efficient algorithms for adding side chains. A test with 35 low resolution density maps shows that the highest ranked structure that has the correct topology can be found within the top 1% of the list ranked by the effective energy that is formed by the helices.

## Methods

**Figure 4:**
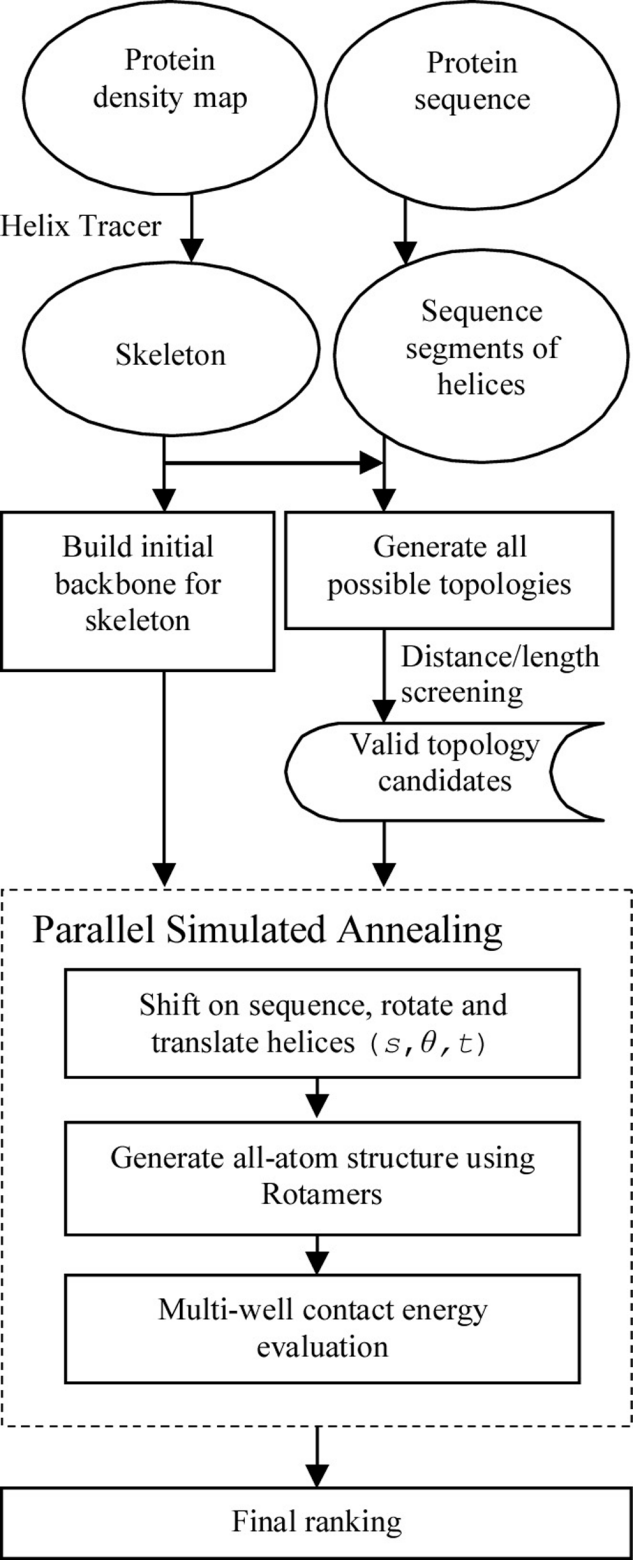
**The structure prediction process for the skeletons**.

The input of the method includes two sources of information: the low resolution protein density map and the sequence of the protein. The density map was simulated from the native 3-dimensional structure of the protein in the PDB to 10 Å resolution using EMAN [33]. Helix Tracer was used to detect the location of helical skeletons in the density map [8]. In order to map the skeletons to their corresponding sequence segments, we generated all the  possible topologies of the skeletons, where *N *is the number of helices in the native protein and *K *is the number of helical skeletons [26] (Figure 4). To eliminate the unlikely topologies in the early stage of the process, a combination of the distance and length screening was conducted. For each possible backbone of the skeleton, the distance, *d*, between the last C atom of a skeleton and the first N atom of the next skeleton is measured. We eliminated the topologies that satisfy *d *> 3.8 (*n*_*loop *_+ 2*s*) where *n*_*loop *_is the number of amino acids on the loop connecting the two adjacent skeletons and *s *is the maximum number of shift allowed in the sequence assignment. We used *s *= 2 for the work in this paper. This rule was used due to the fact that there is a minimum number of amino acids needed to connect two points at a certain distance. The other rule we used to eliminate the bad topologies is to require an equivalent length detected from the skeleton and that from the sequence segment. A skeleton has an equivalent length as a sequence segment if their length difference is within 50% of the length of the skeleton. The length of a helix skeleton is the number of amino acids it contains estimated using a rise of 1.3 Å per amino acid.

Since the secondary structures such as helices and strands have more or less consistent backbone torsion angles, we generated a pool of possible backbone structures that share the same central helix axis. For each of the skeletons, an initial backbone was constructed using the torsion angles (ϕ = -60°, ψ = -50°) to simulate a perfectly straight helix. We then generated an alternative structure by applying a rotation, θ, and a translation, *t*, to the initial backbone of the skeleton around its helix axis. Since each topology determines an assignment between the sequence segments and the skeletons, it is possible to assemble the side chains to the backbone. To simulate the inaccuracy of the secondary structure prediction, we introduced a shift, *s*, for each sequence segment. *S *= *p*_*p *_- *p*_*t *_where *p*_*p *_is the index of the center amino acid of the predicted sequence segment and *p*_*t *_the index of the center amino acid of the helix sequence segment in the native structure. Thus, for each topology, we constructed a pool of backbones, each of which can be represented by a set of parameters (*S*_1_, θ_1_, *t*_1_), (*S*_2_, θ_2_, *t*_2_), ..., (*S*_*k*_, θ_*k*_, *t*_*k*_), when there are *k *skeletons in the density map. For each backbone constructed, we added the side chains based on a specific topology. The side chains were added using the rotamer library and the algorithm of R3 [34-36]. We developed a parallel simulated annealing process to optimize the all-atom structure for the skeletons using a multi-well energy function previously developed [25]. A set of 55 processors were used in a master-slave dynamic load-balance implementation to perform the optimization. The master processor sends topology variables (the orders and the directions) and the set of parameters (*Si*, *θi*) to each available processor. Each slave processor executes a simulated annealing process on the given topology.

## Competing interests

The authors declare that they have no competing interests.

## Authors' contributions

KA developed and implemented the method. WS provided the energy function. JH directed the project and co-developed the methodology.
